# Don't Miss the Warning Signs: Syncope Masquerading as a Major Congenital Heart Defect in a 42-Year-Old Patient

**DOI:** 10.7759/cureus.82098

**Published:** 2025-04-11

**Authors:** Camryn D Rai, Kamal Masri

**Affiliations:** 1 Research, Edward Via College of Osteopathic Medicine, Shreveport, USA; 2 Pulmonary and Critical Care Medicine, Willis Knighton Health System, Shreveport, USA

**Keywords:** atrial septal defect (asd), cardiology, congenital heart defect, partial anomalous pulmonary venous return, syncope

## Abstract

Congenital heart defects (CHDs) are typically diagnosed in infancy, yet some may go unidentified until adulthood and pose significant clinical challenges. We present the case of a 42-year-old female patient with multiple CHDs, including sinus venosus atrial septal defect (ASD), partial anomalous pulmonary venous return (PAPVR), and moderate mitral regurgitation discovered incidentally after presenting to the emergency department with atypical chest pain. Initial warning signs were overlooked, and asymptomatic mitral valve prolapse (MVP) diagnosed in childhood had progressed into moderate mitral regurgitation due to lack of follow-up. Diagnostic workup, including transesophageal echocardiography (TEE), cardiac magnetic resonance imaging (MRI), and catheterization, revealed significant abnormalities requiring surgical intervention. This case highlights the importance of recognizing subtle warning signs, such as palpitations and unexplained fatigue, which may indicate underlying CHDs. Timely diagnosis and management are crucial in mitigating potentially life-threatening complications associated with undiagnosed CHDs in adults. Further research is needed to improve surveillance and treatment strategies for the growing population of adults with CHDs.

## Introduction

It is estimated that 40,000 children born in the United States per year have congenital heart defects (CHD). Of these children, approximately one in four will have a critical heart defect typically requiring intervention in the first year of life [1]. These heart defects are usually detected during newborn screenings or primary care appointments in the first year of life, but the wide variety of presentations can lead to some patients living into adolescence or even adulthood unaware that they possess congenital defects. In fact, it is estimated that approximately 1.4 million adults living in the United States in 2010 had underlying CHDs [2]. Congenital heart defects are a multifactorial process and have been linked to genetic causes such as those leading to syndromes such as Turner syndrome (XO), Down syndrome (trisomy 21), and DiGeorge syndrome (21q11 deletion), as well as neonatal exposure to teratogens such as drugs and medicines such as retinoic acid, viruses such as measles and rubella, and even vitamin deficiencies. There is even some research linking the prevalence of maternal factors such as obesity and age [3,4].

Atrial septal defects (ASDs) are one of the most common types of CHDs. ASDs occur due to malformation of the septum between the right and left atria and are due to failed formation of the ostium premum, ostium secundum, or sinus venosus during embryogenesis. These defects vary in size and may close spontaneously and have no clinical significance or can be the cause of major left-to-right shunting and a predisposing factor for complications such as dysrhythmias and stroke [5]. Anomalous pulmonary venous return is a less common CHD where a patient's pulmonary veins drain into structures such as the right atrium, vena cava, or even coronary arteries instead of the left atrium as expected. This defect can be the cause of left-to-right shunting and is classified as total anomalous pulmonary venous return (TAPVR) or partial anomalous pulmonary venous return (PAPVR). The severity of this defect is generally determined by the percentage of pulmonary venous blood affected [6]. Other CHDs include malformation of cardiac valves such as mitral valve prolapse (MVP), where mitral valve leaflets prolapse into the left atrium due to myxomatous degeneration or fibroelastic deficiency. Mitral valve myxomatous degeneration, also known as Barlow's disease, involves both leaflets of the mitral valve and is commonly associated with connective tissue disorders such as Marfan syndrome, Ehlers-Danlos syndrome, and even polycystic kidney disease [7].

There is vast research on the various types of CHDs, and advancements in surveillance and medical/surgical interventions have dramatically improved the duration and quality of life for children diagnosed with CHDs. However, current research gives minimal attention to adults with CHD. Often, these CHDs have warning signs that can go unrecognized for decades. For example, an ASD may go entirely unrecognized until a patient experiences a thromboembolic stroke or cyanosis due to Eisenmenger syndrome or shunt reversal [5,8]. Patients with PAPVR may be completely asymptomatic, especially if subtle warning signs of pulmonary arterial hypertension (PAH) and right-sided heart failure, such as fatigue, dyspnea, palpitations, and syncope, go unnoticed [9]. While valvular diseases such as MVP can be identified early in the lifespan through identifying a heart murmur, they can also be asymptomatic and worsen over time [7]. Here, we present a case of a patient with a constellation of major CHDs that went undiagnosed until the fifth decade of life and discuss the subtle warning signs that these defects present.

## Case presentation

Here, we present the case of a 42-year-old white female patient who initially presented to the emergency department with complaints of a 10-minute episode of 10/10 retrosternal chest pain radiating to the back relieved by rest that she was concerned was a heart attack. She had a history of anxiety/depression, mitral valve prolapse, and one episode of shingles one year prior to presentation. The patient was a nonsmoker with a body mass index (BMI) of 21.3 kg/m^2^ who reported no alcohol or illicit drug use, with the exception of medical marijuana. Her only allergy was to opioid medications, which caused GI upset. She reported taking no medications at home. As a child, she experienced an episode of syncope during a field day at school, which caused her to see a pediatric cardiologist. At that time, she was diagnosed with asymptomatic mitral valve prolapse, but there were no medical interventions taken, and the patient never followed up. Ultimately, the patient was healthy with an unremarkable medical history.

Upon further questioning, the patient reported worsening fatigue and several recent episodes of near syncope, chest pain, and palpitations. During this visit, the patient presented with a blood pressure of 145/99 mmHg, a pulse rate of 86 beats per minute, and a respiratory rate of 18 breaths per minute with 99% O2 saturation on room air. Initial laboratory tests, electrocardiogram (EKG), and chest X-ray done in the emergency department were unremarkable for any acute process, so the patient was discharged with a referral for outpatient cardiology.

**Figure 1:**
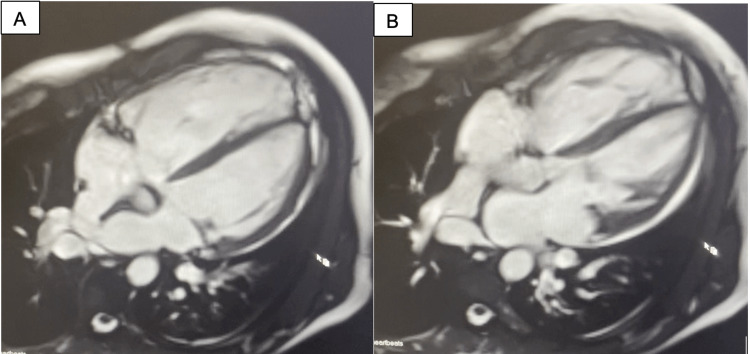
Cardiac MRI showing sinus venosus ASD (A) and partial anomalous venous return of the right upper and middle pulmonary veins into the right atrium (B) MRI: magnetic resonance imaging, ASD: atrial septal defect

She returned to an outpatient cardiology clinic several weeks later with similar symptoms, and an initial transthoracic echocardiogram returned abnormal with signs of mitral regurgitation and right atrial dilation. This concern for right-sided volume overload prompted further investigation, including cardiac magnetic resonance imaging (MRI) (Figure 1), transesophageal echocardiogram (TEE) (Video 1), and right and left heart catheterization (Table 1). This imaging revealed that the patient had a large sinus venosus atrial septal defect (ASD) with partial anomalous pulmonary venous return (PAPVR) of the right upper and middle pulmonary veins draining into the junction of the superior vena cava and right atrium. The patient was found to have a moderately enlarged right atrium and ventricle and WHO class 3 right heart failure with preserved ejection fraction. Additionally, the patient had moderate mitral regurgitation due to myxomatous degeneration of the mitral valve with posterior leaflet prolapse.

**Video 1:** TEE video with Doppler showing flow of pulmonary venous blood into the right atrium TEE: transesophageal echocardiogram

**Table 1:** Hemodynamic studies from right heart catheterization * indicates abnormal values. LVEF: left ventricular ejection fraction, RVEF: right ventricular ejection fraction

Measurement	Patient's value	Reference range
Right atrial pressure (systolic/diastolic/mean)	13/13/9 mmHg*	0-7 mmHg
Right ventricular pressure (systolic/diastolic/mean)	36/4/11 mmHg*	15-25 mmHg in systole, 0-8 mmHg in diastole
Pulmonary arterial pressure (systolic/diastolic/mean)	29/5/14 mmHg*	15-25 mmHg in systole, 8-15 mmHg in diastole
Pulmonary capillary wedge pressure (systolic/diastolic/mean)	15/15/12 mmHg*	6-12 mmHg
O2 saturation in the aorta	98%	95%-100%
O2 saturation in the right atrium	78.1%	72%-86%
O2 saturation in the pulmonary artery	91.5%*	76%
Qp/Qs ratio	3.1*	<1.5: small shunt, 1.5-1.9: moderate shunt, >2.0: large shunt
LVEF	60%-65%	50%-70%
RVEF	63%	50%-70%

Finally, the patient was scheduled for surgery to repair the CHD. She underwent mitral valve repair with posterior band annuloplasty and suture closure of P1/P2 cleft prolapse, closure of the sinus venosus ASD and PAPVR with an autogenous pericardial patch to redirect flow into the left atrium, patching of the enlarged portion of the SVC, and resection of the left atrial appendage. The surgery was successful, and the patient's postoperative course was uneventful. She will continue to be monitored closely for future palpitations and symptoms of heart failure. The patient provided verbal consent for the use of her de-identified information in this report.

## Discussion

One of the most prominent takeaways of this patient's case comes in analyzing the potential warning signs of underlying CHD in adults and how they may have been missed. When this patient was first diagnosed with mitral valve prolapse by echocardiogram, her MVP was mild and had not yet progressed to a degree of mitral regurgitation that would prompt any intervention. Over time, with additional stress on the mitral valve, this progressed to a symptomatic moderate mitral regurgitation. This is a common phenomenon seen in Barlow's disease as the myxomatous degeneration of the mitral valve leads to progressively worsening MVP and eventually mitral regurgitation in adulthood [7]. As MVP is known to progress to mitral regurgitation, it is essential that pediatricians and pediatric cardiologists emphasize the importance of routine follow-ups and monitoring of MVP to implement appropriate interventions before the valvular disorder becomes symptomatic.

During this patient's initial echocardiogram during childhood, it is likely that the lack of enlargement of the right atrium as well as possible limited scope of imaging, limitations in imaging technology at the time, and/or lack of color Doppler contributed to failed early identification of her PAPVR and other abnormalities. In fact, it is common for PAPVR defects to have no clinical manifestations, especially when the return constitutes <50% of total pulmonary venous blood [9]. PAPVRs can exist in the context of an ASD or without. For PAPVR with an ASD, it is most common for a sinus venosus defect to occur with a right-sided venous anomaly, as seen in this patient. However, ostium secundum ASDs were associated with both left- and right-sided PAPVRs [6]. This patient's right-sided PAPVR eventually led to increased pressure in the right side of the heart and eventual right atrioventricular enlargement on echocardiogram, prompting further investigation. For patients who have PAPVR without an associated ASD, right atrioventricular dilation and associated pulmonary arterial hypertension are more likely clinically significant [10]. For many patients with unrepaired PAPVR, the presenting symptoms and subsequent diagnosis are due to pulmonary arterial hypertension (PAH). PAH may be difficult to diagnose as it typically presents with nonspecific symptoms such as dyspnea, cough, and fatigue [10]. Interestingly, this patient did not have significant pulmonary arterial hypertension with a pulmonary arterial pressure of 29/5/14 mmHg (normal: 15-25 mmHg in systole and 8-15 mmHg in diastole). It is likely that the patient's large ASD (Qp/Qs: 3.1) contributed to right-sided volume overload and right chamber dilation, which further mitigated the pulmonary arterial hypertension and delayed any associated symptoms.

One of the most prominent warning signs leading to this patient's diagnosis of CHD was palpitations. It was later discovered that this patient was having episodes of atrial tachycardia, likely due to stretching and fibrosis of the right atrium and the underlying cardiac electrical system. The presence of atrial fibrillation in adults with CHD is thought to be 10%-30%, with a 50% lifetime risk of atrial arrhythmia in adults with CHD. These atrial arrhythmias are particularly associated with malformations of the atria, such as ASD and ectopic pulmonary veins [11]. In this patient, episodes of palpitations and chest pain were important warning signs leading to her eventual diagnosis of CHD. However, it is important to note that this patient's previous history of anxiety may have masked some of these symptoms and further prolonged her diagnosis. Adults with CHD have a significantly increased risk of experiencing mental health disorders such as anxiety and depression for a variety of reasons. This makes it increasingly important to prioritize mental health interventions for patients with CHD and identify symptoms such as palpitations and dyspnea in patients with complex mental health history as potential warning signs for underlying CHD [12,13].

## Conclusions

Ultimately, this case brings to light the importance of identifying and further investigating warning signs of underlying CHDs in adults as well as diligently following up on asymptomatic cardiac abnormalities identified during childhood. For this patient, the underlying CHD led to a constellation of nonspecific symptoms such as syncope, dizziness, palpitations, and fatigue that were ultimately attributed to the patient's CHDs. Each of these symptoms served as nonspecific warning signs that eventually prompted further investigation. More research is needed in the field of identification and management of CHDs in adults to promote prompt diagnosis and treatment of potentially fatal heart conditions.
